# Supplementary material for: Evidence of phylosymbiosis in Formica ants
Source: Front Microbiol. 2023 May 5;14:1044286. doi: 10.3389/fmicb.2023.1044286 (PMC10196114; doi:10.3389/fmicb.2023.1044286)
Supplement: Supplementary file 1 [file Data_Sheet_1.docx]

Supplementary Materials - Images


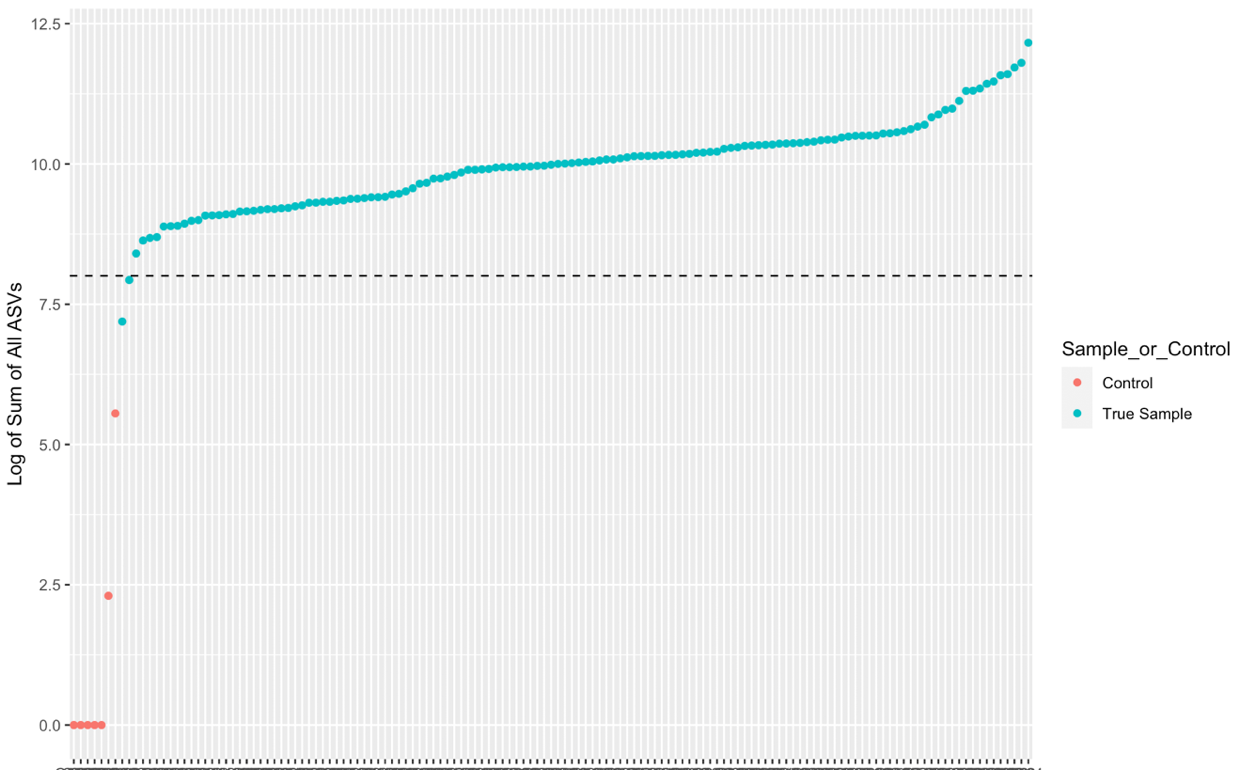


Figure SI1: Total ASVs Measured in Each Sample. Samples are coloured by whether they are control samples. Order on the x-axis is sorted by number of ASVs. The dotted line represents the chosen cutoff for the minimum number of ASVs present to be considered a successfully sequenced sample.


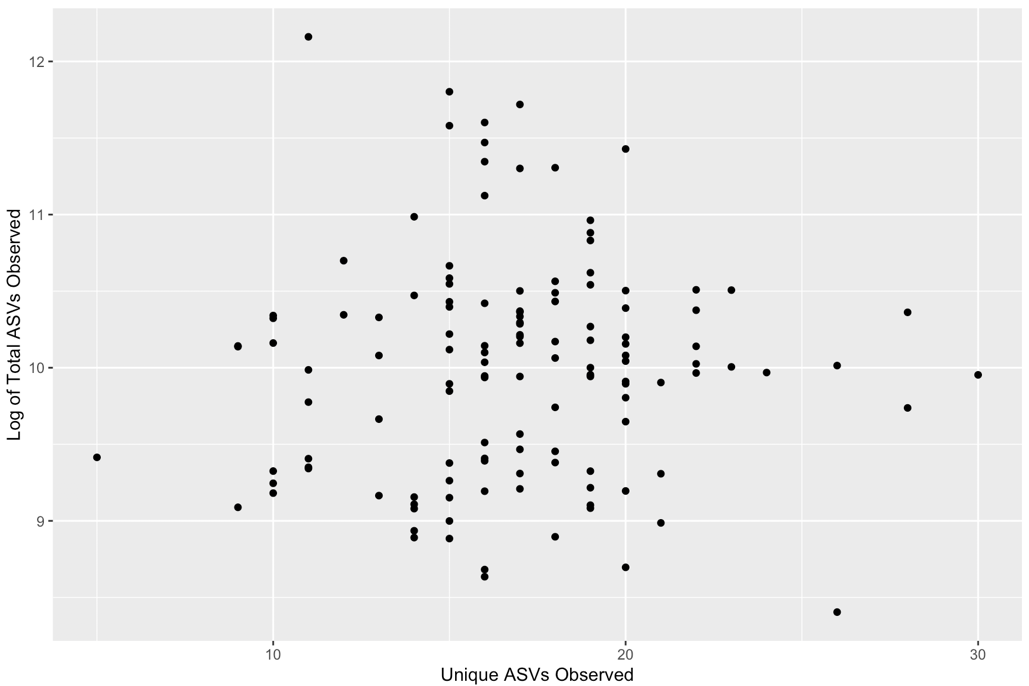


Figure SI2: Total ASVs measured per sample versus unique ASVs measured per sample. This relationship was modelled using a generalized linear model from the quasipoission family, which found no significant effect of log of Total ASVs observed on number of unique ASVs observed. The model was run as glm (Unique_ASV ~ log(Total_ASV+1)). The t-value of log(Total_ASV+1) was 0.427 with a p-value of 0.67. Null deviance was 130.37 on 129 degrees of freedom. Residual deviance was 130.19 on 128 degrees of freedom.
