# Supplementary material for: Evidence of phylosymbiosis in Formica ants
Source: Front Microbiol. 2023 May 5;14:1044286. doi: 10.3389/fmicb.2023.1044286 (PMC10196114; doi:10.3389/fmicb.2023.1044286)
Supplement: Supplementary file 2 [file Data_Sheet_2.docx]

Supplementary Materials

**Figure S1: Full Phylogeny Generated by Silva ACT**

Names consist of species designation, isolation source, and either standardized ID or OTU ID. OTU names end with their specified abbreviation.
